# Phenotypic variation in *Blastocystis* sp. ST3

**DOI:** 10.1186/1756-3305-7-404

**Published:** 2014-08-29

**Authors:** Nanthiney Devi Ragavan, Suresh Kumar Govind, Tan Tian Chye, Sanjiv Mahadeva

**Affiliations:** Department of Parasitology, Faculty of Medicine, University of Malaya, Kuala Lumpur, 50603 Malaysia; Department of Medicine, Faculty of Medicine, University of Malaya, Kuala Lumpur, 50603 Malaysia

**Keywords:** *Blastocystis*, Phenotypic, Subtype 3, Pathogenicity

## Abstract

**Background:**

*Blastocystis,* is one of the most common human intestinal protozoan, which has many conflicting reports on its pathogenic role. Gut conditions which obviously varies in asymptomatic individuals, symptomatic and irritable bowel syndrome (IBS) patients in terms of gut flora, pH, osmotic pressure and water potentials could play an important role in its pathogenicity. The present study is the first study to investigate phenotypic characteristics of *Blastocystis* sp. ST3 isolated from asymptomatic, symptomatic and IBS isolates.

**Methods:**

A total of 8 *Blastocystis* isolates were obtained from four IBS patients (IBS1-4) and four symptomatic patients (S1-4) at a local gastroenterology clinic. Asymptomatic isolates (A1-4) were obtained from a field survey at a local village.

**Results:**

All 12 isolates were determined as subtype 3 (ST3). A1-4 isolates showed the highest peak growth followed by IBS1-4 isolates and S1-4 isolates for the growth profile. Parasites from IBS isolates (IBS1-4) showed the largest diameter with a mean 18.43 ± 2.22 μm compared to parasites of symptomatic isolates (isolates S1-4) 15.54 ± 3.02 μm and asymptomatic isolates (isolates A1-4) 11.76 ± 0.82 μm. The symptomatic isolates (average generation time: 9.87 ± 2.97 h) grew faster than the IBS isolates (average generation time: 7.56 ± 1.06 h) and asymptomatic isolates (average generation time: 5.97 ± 1.52 h). The parasites isolated from IBS isolates showed strong aggregation and clumping, which had seen reduced in parasites of isolates S1-4. No clumping was seen in parasites from A1-4. The outer surface of parasites in IBS isolates showed greater binding affinities towards FITC-labelled Concanavalin A (Con A) than symptomatic isolates and asymptomatic isolates. Scanning electron microscopy showed that in IBS isolates, the surface of *Blastocystis* showed a very coarse and intensely folded surface. The IBS isolates also exhibited a dense material and a thicker layer of surface coat can be seen compared to asymptomatic and symptomatic isolates.

**Conclusion:**

There have been no studies thus far providing evidence for phenotypic variation within a particular subtype. The present study is the first to demonstrate the phenomenon of gut environment facilitating adaptation of parasites possibly for survival leading to phenotypic differences for Blastocystis*.*

## Background

*Blastocystis,* the most common human intestinal protozoan [1], has the fecal-oral route as the usual mode of transmission with its infective stage being the cyst form [2]. Although clinical signs of *Blastocystis* infection have been shown to be mainly diarrhoea, abdominal pain as well as non-specific gastrointestinal symptoms such as nausea, anorexia, vomiting, weight loss, lassitude, dizziness, and flatulence [3], however the conflicting reports on its pathogenic role still continues with the organism reported in many healthy individuals who don’t exhibit any symptoms.

Phenotypic differences between symptomatic and asymptomatic isolates have been also shown through isoenzyme patterns [4, 5], protein profiling and [6, 7] sero-groups [8] and molecular characterization [9–16]. A recent study reported that solubilized antigen of *Blastocystis* (Blasto-Ag) derived from symptomatic isolates was more pathogenic and possess the ability to weaken the cellular immune response compared to the asymptomatic isolates [17].

There have been 13 subtypes reported [15] with *Blastocystis* ST3 shown to be the highest in Thailand with prevalence rates between 41.7-92.3% [18]; Egypt-54.55% [19]; Singapore- 78% [20]; Turkey- 75.9% [21]; Turkey-59.3% [22]; Germany- 21% [9] and France- 53.5% [23]. These studies demonstrate the importance of subtype 3 in terms of its prevalence and its pathogenic implications.

*Blastocystis* had been associated with irritable bowel syndrome (IBS) [24] and the consequence of gut conditions which obviously varies in asymptomatic individuals, symptomatic and IBS patients in terms of gut flora, pH, and osmotic pressure and water potentials has never been investigated. It is obvious that the severity would be obviously seen more in IBS than in the gut conditions of symptomatic or asymptomatic condition [25].

We fortuitously through field and other clinical surveys over a length of time collected subtype 3 *Blastocystis* from three different groups, asymptomatic individuals, symptomatic and IBS patients and in the present study attempt to evaluate if the parasite from these three different gut conditions can influence phenotypic variation of the parasite.

## Methods

### Source of *Blastocystis*isolates

A total of 8 *Blastocystis* isolates were obtained from four IBS patients (IBS1-4) and four symptomatic patients (S1-4) at a local gastroenterology clinic. Asymptomatic isolates (A1-4) were obtained from a field survey at a local village. Irritable bowel syndrome was defined according to the Rome III criteria [26] whereas gastrointestinal symptoms which varied from stomach bloating, diarrhoea, abdominal cramp to excessive gas. Stool screening were also done for various intestinal parasites such as *Entamoeba histolytica, Giardia lamblia,* microsporidia*, Dientamoeba fragilis, Ascaris lumbricoides, Trichuris trichiura,* hookworm and *Taenia sp.* Patients who were positive for one or more of these parasites was excluded from the present study.

The parasites were isolated by culturing in 3 ml Jones’ medium supplemented with 10% horse serum (Gibco Laboratories, Life Technologies) and maintained at 37°C. The positive cultures were sub-cultured once every three days and also stored at -20°C for subsequent subtyping. Consequently after isolation, the parasites were maintained in Jones’ medium supplemented with 10% horse serum for at least one month prior to the elucidation of phenotypic characteristics.

### Subtyping of *Blastocystis*

The genomic DNA of *Blastocystis* for all 12 isolates were extracted using QIAamp DNA stool mini kit (Qiagen, Hilden, Germany) according to the manufacturers’ protocol. All 12 *Blastocystis* isolates were then subjected to sequence tagged site (STS) primer-polymerase chain reaction (PCR) using the seven sets of primers previously described by Yoshikawa *et al*. [18]. Two to five microliters of DNA preparations were used to amplify the genomic sequences in a 20 μl reaction containing 1× PCR buffer (Fermentas, USA). PCR condition consisted of 1 cycle of initial denaturing at 95°C for 5 minutes, followed by 40 cycles of denaturing at 95°C for 1 minute, annealing at 56.3°C for 1 minute 30 seconds and extending at 72°C for 1 minute, and an additional cycle of elongation at 72°C for 10 minute (Thermocycler Bio-Rad). The amplified products were then electrophoresed in 1.5% agarose gels (Promega, USA) in Tris–borate-EDTA buffer. Gels were stained with ethidium bromide and photographed using an ultraviolet gel documentation system (Uvitec, United Kingdom). PCR amplification for each primer pair were done in triplicate.

### Growth characteristics of *Blastocystis*

The parasites of each isolate were pooled together from day 3 cultures to make a final concentration of 1x10^4^*Blastocystis* per ml in 3 ml screw-capped tubes containing Jones’ medium supplemented with 10% horse serum. All cultures were kept in airtight screw- capped tubes and incubated at 37°C for up to 10 days. All experiments were done in triplicates. The *Blastocystis* count was carried out using haemocytometer chamber (Improved Neubauer, Hausser Scientific) with 0.4% trypan blue dye exclusion (Sigma-Aldrich Corp. USA) as viability indicator. The parasite count was determined daily in cultures until it became non-viable. Only viable cells that did not take up trypan blue stain were counted.

Fifty parasites were randomly chosen from every culture tube for size measurement every 2 day culture period for the next 10 days. Statistical analysis was carried out using SPSS version 21. Generation time (GT) was calculated for the 24-h period during the most rapid growth based on the following formula as described by Chaudhari and Singh [27].


### Modified fields’ stain

The *Blastocystis* isolates from day 3 culture were stained with Modified Fields’ stain according to the protocol by Afzan [28]. The slides were then viewed under 400X magnification. All experiments were done in triplicates.

### Cytochemical staining

The parasites were then grown, pooled and centrifuged respectively to make smears on day 3 cultures with Fluorescein isothiocyanate (FITC)-labelled Con A (*Canavalia ensiformis*) [16] and examined with a fluorescence microscope (LeitzWetzlar, Germany) using incident light transmission at 400X magnification. All experiments were done in duplicate. The results were then quantified by using percentage of fluorescent cells in 100 cells and affinity fluorescence unit (AFU) (scale of brightness of 1+, 2+, 3+ and 4+).

### Scanning electron microscopy

The parasites were washed three times with phosphate buffered saline (PBS) pH 7.4. The samples were centrifuged at 2000 × g for 5 min. The pelleted cells were fixed with 4% glutaraldehyde and post-fixed with 1% osmium tetroxide. The specimens were then mounted on polycarbonate membrane (Nuclipore, Agar Scientific, USA) and dehydrated in increasing concentrations of ethanol (30%, 50%, 70%, 80%, 90% and 100%). The specimens were critical-point dried with carbon dioxide coated with gold, and examined with a scanning electron microscope (FEI-Quanta 200 FESEM, USA).

### Transmission electron microscopy

Culture containing *Blastocystis* was collected from day 3 culture washed three times using PBS pH 7.4 and centrifuged at 2000× g, for 5 min. The pelleted cells were re-suspended overnight in 4% glutaraldehyde in 0.1 M sodium cacodylate buffer, pH 7.3 at 4°C, washed thoroughly with cacodylate buffer and post-fixed for 30 min in 1% osmium tetroxide in cacodylate buffer. The fixed cells were dehydrated for 5 minutes in ascending series of ethanols (30%, 50%, 70%, 80%, 90% and 100%) and embedded in epoxy resin. Semithin sections were stained with toluidine blue. Ultrathin sections were cut using an ultramicrotome, contrasted with uranyl acetate and lead citrate and viewed using a transmission electron microscope (LEO Libra120).

## Results

### Genotyping of *Blastocystis*

**Figure 1:**
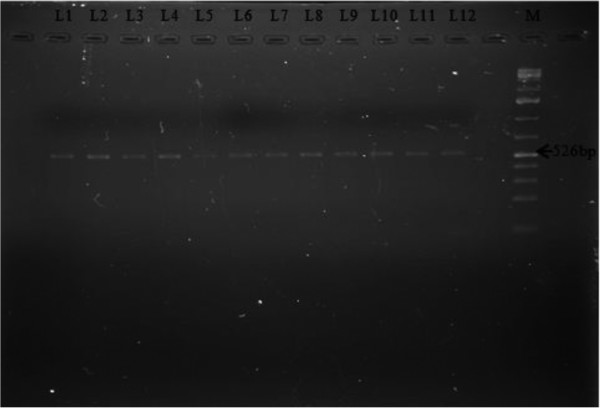
**Genotyping of** ***Blastocystis*** **isolates isolated from asymptomatic individuals L1-L4, symptomatic isolates L5-L8, and IBS isolates L9-12, ST 3 (526 bp), M = 100 bp plus DNA marker.**

Based on PCR amplification with the STS primers, all the 12 isolates from 3 different groups, asymptomatic, symptomatic and IBS isolates amplified with the primer SB227 (~526 bp) [18] (Figure 1). The PCR products for all three groups were determined as subtype 3 (ST3). In this study, we found that subtype 3, which also known as pathogenic subtype is also found in the asymptomatic isolates.

### Growth characteristics

**Figure 2:**
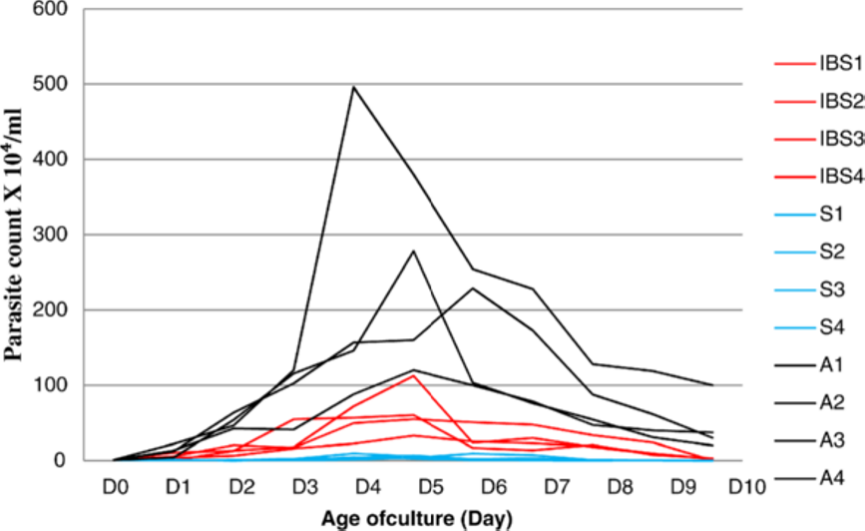
**Growth profile of** ***Blastocystis*** **isolates A1-4, IBS1-4 and S1-4 in Jones’ medium supplemented with 10% horse serum.**

**Figure 3:**
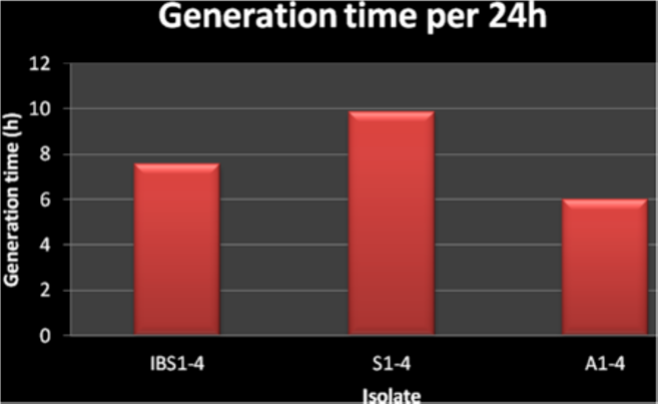
**Comparison between generation time for IBS isolates (IBS1-4), symptomatic isolates (S1-4) and asymptomatic isolates (A1-4) of** ***Blastocystis*** **isolates.** Note that the asymptomatic isolates grew faster than both IBS isolates and symptomatic isolates.

**Figure 4:**
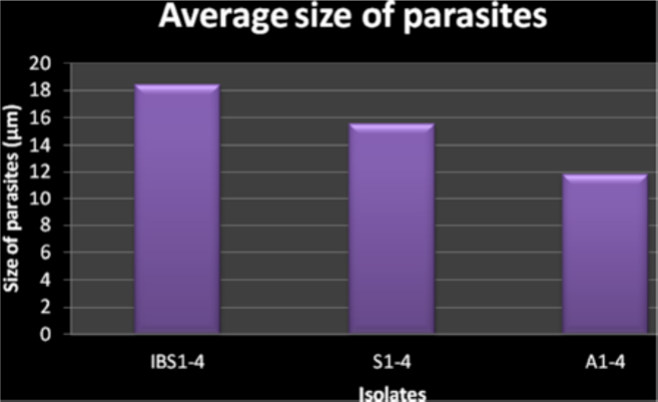
**Comparison between average size of parasites for IBS isolates (IBS1-4), symptomatic isolates (S1-4) and asymptomatic isolates (A1-4) of** ***Blastocystis*** **isolates.**

A total of 12 *Blastocystis* ST3 isolates from four IBS patients (IBS1-4), symptomatic patients (S1-4) and asymptomatic individuals (A1-4) respectively were used in the present study. The growth profile with an initial inoculation of 1.0X10^4^ cells/ml showed three distinct and different growth profiles. A1-4 isolates showed the highest peak growth compared to the other two groups followed by IBS1-4 isolates and S1-4 isolates. The range of parasite count during peak growth in IBS isolates was between 33.1X10^4^ cells/ml to 112.4X10^4^ cells/ml (Figure 2) and in symptomatic isolates between 4.53X10^4^ cells/ml to 9.33X10^4^. The parasite peak count for A1-4 isolates ranged from 120X10^4^ cells/ml in isolate A1 to 496X10^4^ cells/ml in isolate A2(Figure 2). All IBS isolates peaked on day 5 including one and two of the symptomatic and asymptomatic isolates respectively. The remaining isolates peaked on day 4 with the exception of isolates S2 and A4 which peaked on day 6 (Figure 2).The symptomatic isolates (average generation time: 9.87 ± 2.97 h) grew faster than the IBS isolates (average generation time: 7.56 ± 1.06 h) and asymptomatic isolates (average generation time: 5.97 ± 1.52 h) (Figure 3). Parasites from IBS isolates (IBS1-4) showed the largest diameter with a mean of 18.43 ± 2.22 μm compared to parasites of symptomatic isolates (isolates S1-4) 15.54 ± 3.02 μm and asymptomatic isolates (isolates A1-4) 11.76 ± 0.82 μm (Figure 4).

### Modified fields’ stain

**Figure 5:**
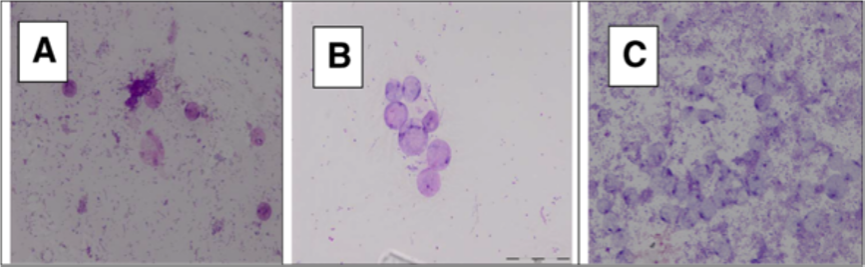
**Comparison of clumping of** ***Blastocystis stained with modified field stain*** **for IBS isolates (IBS1-4), symptomatic isolates (S1-4) and asymptomatic isolates (A1-4) of** ***Blastocystis*** **isolates** **.** **A**: A1 isolate were stained with modified field stain and the parasites were far apart; **B**: Blastocystis from S2 isolates were attached together in a smaller number of parasite; **C**: The IBS1 isolate seen to be clumped together in a large number.

The staining characteristics in parasites from all three groups were similar. However parasites from IBS isolates showed strong aggregation and clumping (Figure 5C), the intensity of which was markedly seen reduced in parasites of isolates S1-4 (Figure 5B). Parasites from A1-4 isolates were seen to be distinct with no clumping (Figure 5A).

### Cytochemical staining of *Blastocystis*

**Figure 6:**
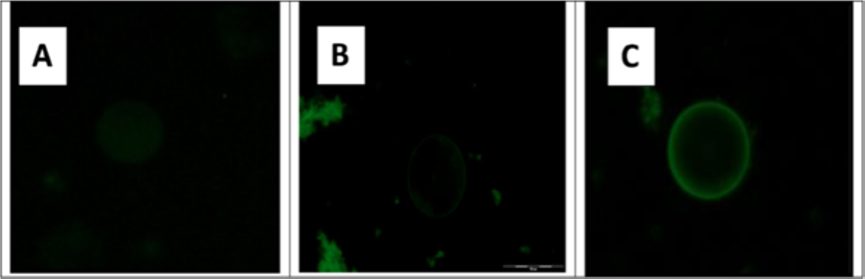
**Comparison of binding affinities of** ***Blastocystis stained with*** **FITC-labelled Con A** ***for*** **IBS isolates (IBS1-4), symptomatic isolates (S1-4) and asymptomatic isolates (A1-4)** ***.*** **C**: IBS isolates (IBS1-4) which were stained with FITC-labelled Con A showed a greater binding affinities with scale brightness of AFU (4+), **B**: symptomatic isolates, isolates S1-4, AFU (3+) and **A**: asymptomatic isolates, isolates A1-4, AFU (1+). The values are expressed as: AFU 1+ weak intensity, AFU 3+ medium intensity, AFU 4+ strong intensity (percentage of reactive forms).

The outer surface of parasites in IBS isolates (Figure 6C) showed greater binding affinities towards FITC-labelled Concanavalin A (Con A) than symptomatic isolates (Figure 6B) and asymptomatic isolates (Figure 6A). The fluorescence intensity and the percentage of the reactive forms of IBS isolates in FITC-labelled Con A stain range was (4+; 88-100%), symptomatic isolates, S1-4 (3+; 78-100%) and for asymptomatic isolates, A1-4 (1+; 78- 94%).

### Surface structure of *Blastocystis*

**Figure 7:**
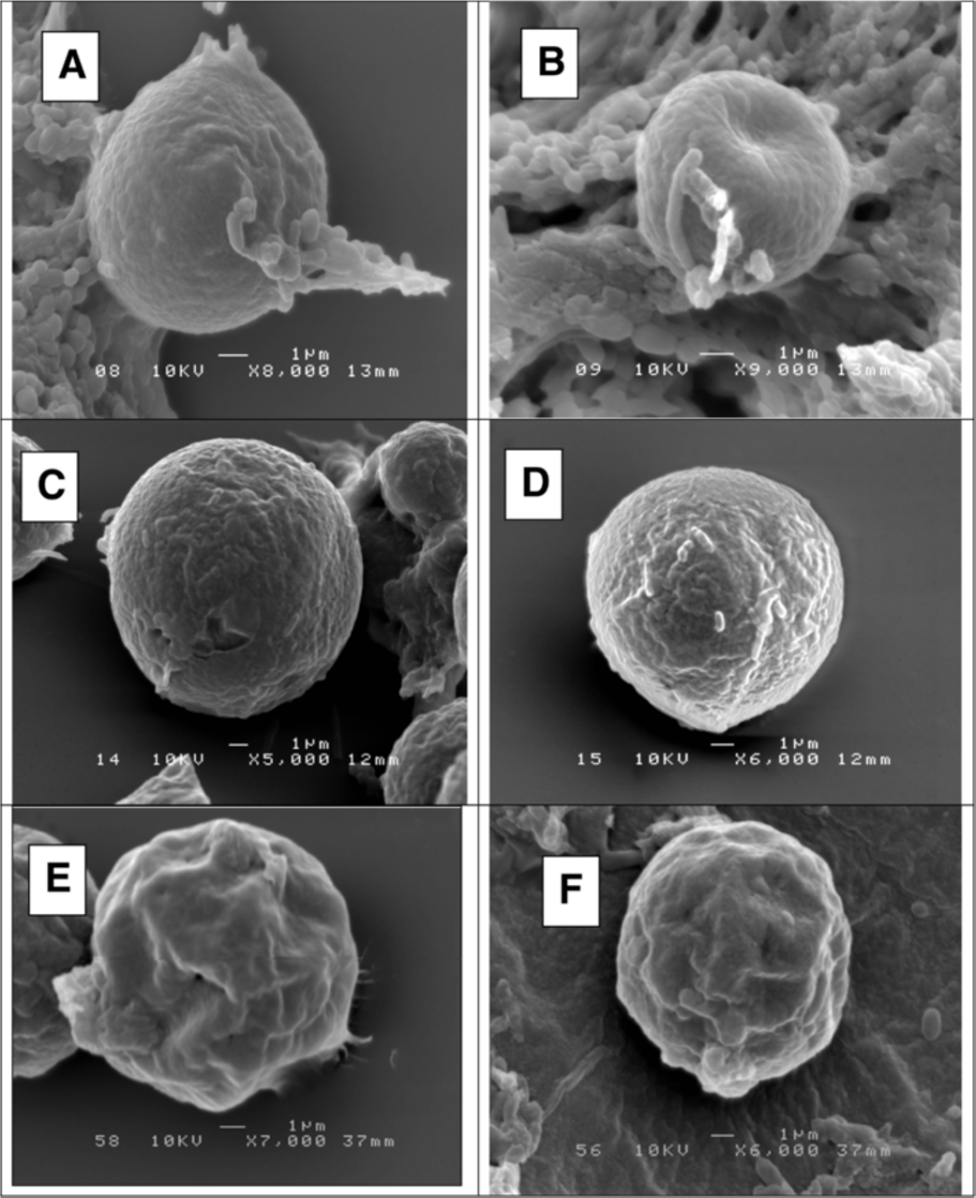
**Comparison of Scanning electron microscopy of** ***Blastocystis for*** **IBS isolates (IBS1-4), symptomatic isolates (S1-4) and asymptomatic isolates (A1-4).** **A** &**B**: Scanning electron microscopy showed that Blastocystis isolated from A1 possess a smooth surface; **C** &**D**: The Blastocystis isolated from symptomatic isolate, S1 showed slightly rough surface; **E** &**F**: The Blastocystis isolated from IBS1 showed coarse and folded surface.

*Blastocystis* forms in three different groups, asymptomatic isolates (Figure 7A&B), symptomatic isolates (Figure 7 B&C) and IBS isolates (Figure 7E&F) appeared to have different surface respectively. Scanning electron microscopy showed that *Blastocystis* isolated from asymptomatic isolates possess a very smooth surface meanwhile the *Blastocystis* isolated from symptomatic isolates showed slightly rough surface with tiny pores. In IBS isolates, the surface of *Blastocystis* showed a very coarse and intensely folded surface.

### Ultrastructure of *Blastocystis*

**Figure 8:**
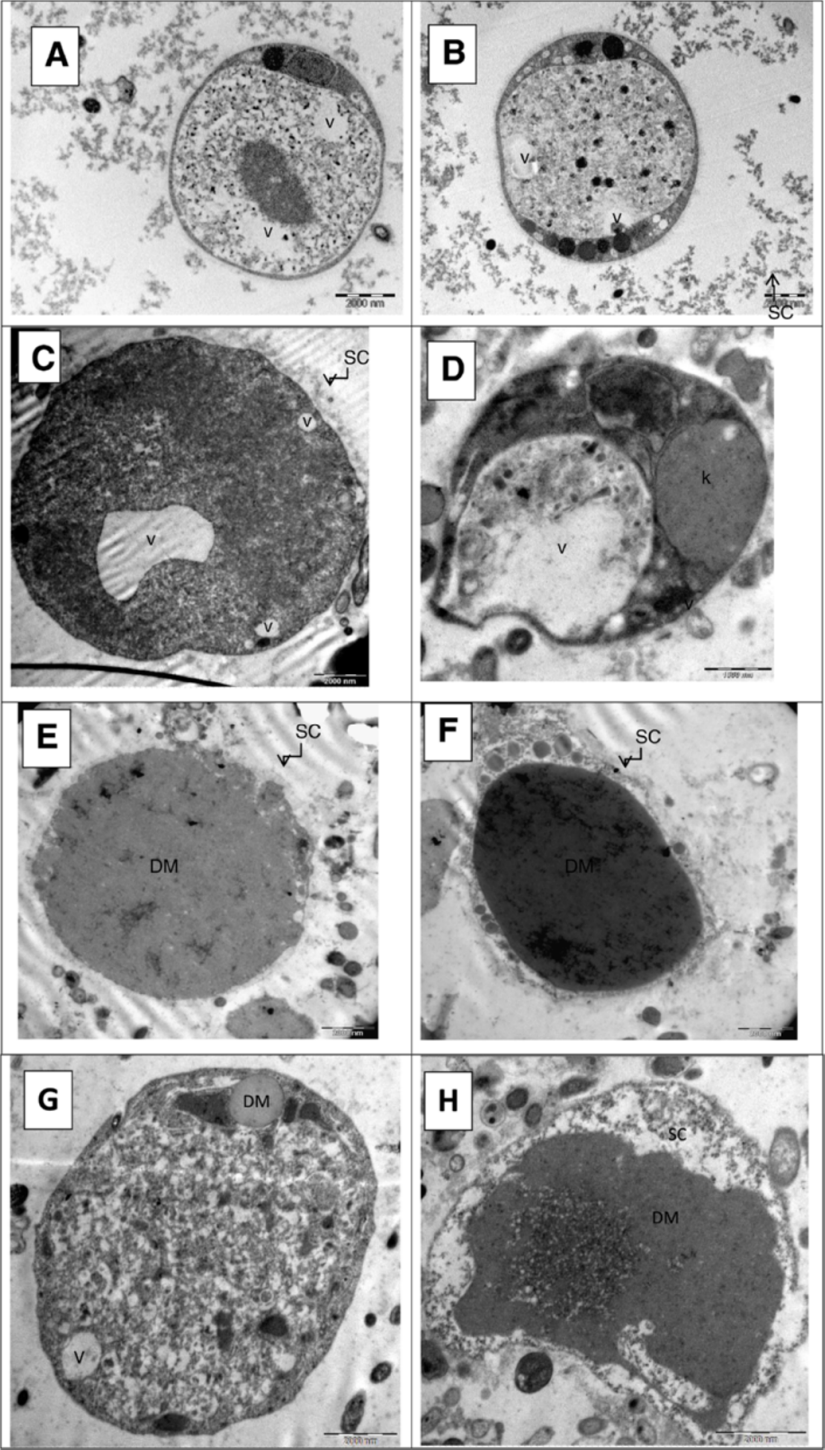
**Comparison of Transmission electron microscopy** ***for*** **IBS isolates (IBS1-4), symptomatic isolates (S1-4) and asymptomatic isolates (A1-4). A**: Transmission electron micrographs (TEM) shows asymptomatic isolates, isolate A1; **B**: The Blastocystis isolated from symptomatic isolates, S1. **C-H**: Electron dense material can be seen within central body of all IBS isolates. Note: (V) vacuole, (DM) dense material, (SC) surface coat.

*Blastocystis* in all three groups, asymptomatic isolates (Figure 8A), symptomatic isolates (Figure 8B) and IBS isolate (Figure 8C-H) appeared to be rounded and oval shape. The IBS isolates also exhibited a dense material (Figure 8C-H). In contrast, the dense material was not seen in any of the parasites from both the asymptomatic and symptomatic isolates.

**Figure 9:**
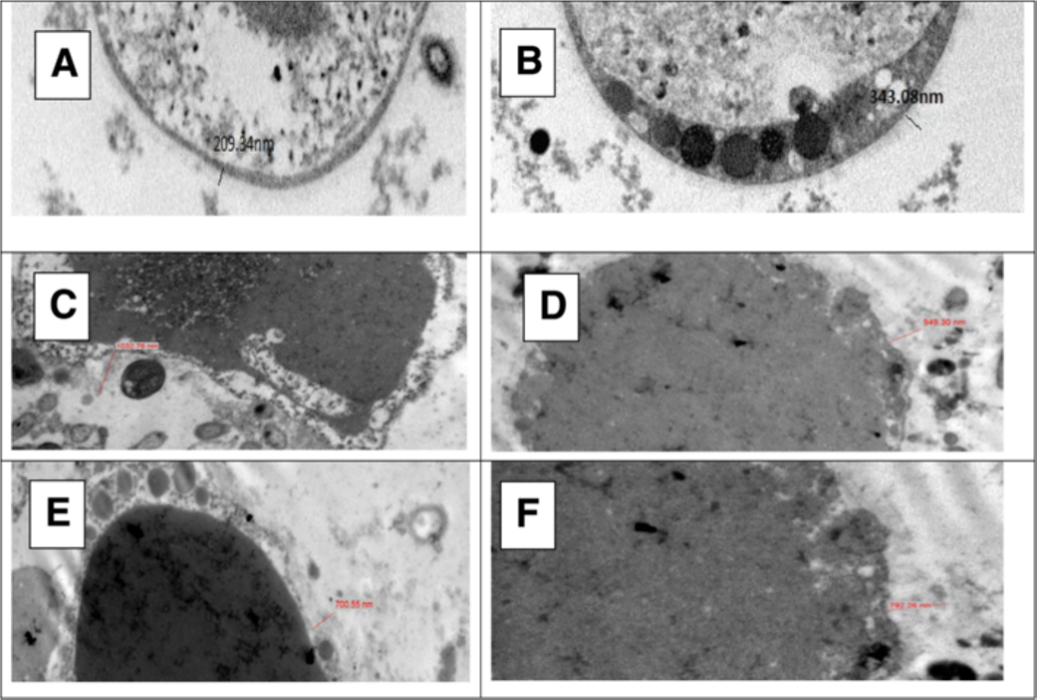
**Comparison of thickness of surface coat for** **IBS isolates (IBS1-4), symptomatic isolates (S1-4) and asymptomatic isolates (A1-4);** ***Blastocystis*** **in IBS isolates (Figure 9C-F) revealed to have a thicker layer of membrane surrounding the parasites which is also known as surface coat compared to a relatively thin layer of membrane in both asymptomatic isolates and symptomatic isolates (Figure 9A& B).**

*Blastocystis* in IBS isolates (Figure 9C-F) showed a thicker layer of surface coat surrounding the parasites compared to a relatively thinner layer seen in both asymptomatic isolates (Figure 9A) and symptomatic isolates (Figure 9B).

## Discussion

The pathogenicity of *Blastocystis* is still disputed because of its presence in both asymptomatic individuals and symptomatic patients [12, 29]. Out of 15 subtypes (ST1-ST15) based on the small subunit rRNA, nine of it had been found in human (ST1-ST9) [30].

Previous studies have demonstrated that parasites from symptomatic and asymptomatic isolates have shown phenotypic differences [16, 17] but these differences could have been largely due to subtype differences. In the present study, the fortuitous discovery of subtype 3 *Blastocystis* from three different groups namely IBS, symptomatic and asymptomatic individuals have been exploited to assess if differing gut conditions can influence phenotypic differences.

Growth profiles have been used previously to demonstrate phenotypic differences [16] and in the present study three distinct growth profiles and generation time were seen for symptomatic, asymptomatic and IBS isolates providing evidence that the rate and possibly the biology of reproduction could be influenced by gut conditions. The generation time shows that the asymptomatic isolates grew faster than symptomatic and IBS isolates which were similar with a study done by Tan et al. [16]. Furthermore the IBS isolates are larger in diameter compared to parasites from symptomatic and asymptomatic. What is interesting to note is that the size variation in IBS isolates is the highest compared to the other two groups implying that the growth conditions in IBS gut would have conferred this diversity in size, growth rate and generation time. There was a notable clumping of parasites seen in all isolates from IBS which was absent totally in all asymptomatic isolates and partially seen in symptomatic isolates. The surface coat of parasites from IBS isolates has been shown to be folded and coarse as seen by SEM and its surface coat seen more prominently thicker by TEM studies. It is highly likely that this surface could be sticky which has resulted in the clumping of the parasites which is seen in the fields’ stain. The surface coat also showed a high Con A binding with 88-100% of cells in all IBS isolates showing affinity fluorescence unit (AFU) of 4+ compared to symptomatic and asymptomatic isolates which showed only 3+ and 1+ respectively.

The digestive system’s purpose is to facilitate the breakdown and absorption of nutrients into the bloodstream. The environment has to be supplemented with the right gut flora [31], pH [32] and the right amount of secretion of acid, bile and pancreatic juices [33]. The pH of gastric acid and small intestine is between pH 1.5 to 3.5 and pH 7.0 to 9.0 respectively. Recent studies have moved the belief that IBS is a purely psychological disorder. There has been clear demonstration of distinct abnormalities of the gut mucosa including immune activation, increased release of inflammatory mediators and impaired gut barrier function. It is obvious that the gut conditions vary between asymptomatic to symptomatic conditions with prominent differences such as low grade inflammation, abnormal gases, immunological response, low fecal microbiota acid level with poorer protein and carbohydrate metabolism in IBS patients [34]. Furthermore the gut diversity with increased bacteria has been reported to be seen within the mucus layer sometimes in the deeper portions previously reported in IBS patients using the fluorescent in situ hybridization method possibly due to a common barrier defect within mucosal layers [35, 36]. Significant difference in CD3+, CD8+, CD4+, LPL and IEL between PI-IBS patients and healthy controls have been shown providing evidence that there has been aberrant mucosal immune response to the luminal environment in PI-IBS [37]. The severity in IBS conditions and the influence of these adverse changes in gut condition obviously seem to have a pronounced effect on the phenotypic expression of *Blastocystis* seen in the present study.

Studies have implicated that genotypes of *Blastocystis* to be the influencing factors for the parasite’s pathogenicity especially subtype 3 where in Malaysia [16] Singapore [20] and USA [38] evidence for its pathogenicity was clearly demonstrated. *Blastocystis* ST3 was also found to be the only subtype in 100% of patients suffering from urticaria in Egypt [39] Blasto-Ag of ST3 showed the most predominant subtype compared to five other subtypes in triggering a higher proliferation rate in colorectal cancer cells [40]. Tan et al. [16] also demonstrated ST3 to be pathogenic when comparing the phenotypic characteristics between symptomatic and asymptomatic isolates of *Blastocystis*.

The present study however is the first to demonstrate phenotypic variation within a subtype 3 population from three environments namely asymptomatic, IBS and symptomatic. Hence environment adaptability for survival purposes could be a possible reason for the phenotypic variation to exist.

The thicker surface coat shown by the ultrastructural study in IBS isolates could influence cytopathic effect of *Blastocystis* towards the intestinal lining of the gut. *Blastocystis* lysate and live parasite have been shown to trigger cytopathic effect on Chinese Hamster Ovary cells [41, 42]. The coarse and uneven surface with folding seen in parasites of IBS isolates showing high Con A binding as well the aggregation and clumping seen in stained smears provide evidence that the surface is sticky. This becomes ideal to facilitate adherence to bacteria [43] as well as to the intestinal lining which can exacerbate inflammation.

In IBS condition inflammation of intestinal layer could cause leaky gut syndrome which facilitates undigested food substances to pass between the cells of the intestinal epithelial layer to the bloodstream causing damage on the intestinal wall [44] which in turn will reduce the efficiency of nutrient absorption. Previous studies have shown that the interplay between luminal factors such as food, living bacteria in the intestine, epithelial barrier and the mucosal immune system can alter structural rearrangement of tight junction proteins in the small intestine and colon which can result in the increasement of intestinal permeability especially in post-infectious IBS and in IBS with diarrhoea [45]. Dietary intake could also influence the intestinal gut flora causing a digestive problem which then can lower the immune system [46]. Other than that, a studies done to evaluate the role of single nucleotide polymorphisms (SNPs) for interleukin (IL)-8 and IL-10, comparing between IBS patients infected with Blatoscytsis and asymptomatic patients found out that it can actually alter individual sensitivity increasing the relative risk in the development of *Blastocystis* infected IBS patients [47].

The present finding has a very important implication. Previously all evidences have been pointing to subtype 3 to be the pathogenic one. However the present study cautions on forming such a conclusion and suggest that ascribing subtype to pathogenicity could be an over generalization. It is evident that gut environment can influence phenotypic expression of even the same subtype. A similar study had been done on assessing the phenotypic characterization of entamoeba hystolytica whereby the interaction between the parasites and host component such as bacterial flora and mucins can trigger the parasites to be more pathogenic by exhibiting their virulences factor [48].

It is beyond the scope of the present study to postulate the details of the gut environment although others have suggested that an unhealthy gut has a complex open ended ecosystem which can be a host for various microorganisms [49–51]. These and other factors had made the difference. The study has diagnostic implications and it is important to ensure that a proper and a detailed study be undertaken before forming any definite conclusion as gut environment does play a part in expressing and influencing phenotypic.

## Conclusion

There have been no studies thus far providing evidence for phenotypic variation within a particular subtype. The present study is the first to demonstrate the phenomenon of gut environment facilitating adaptation of parasites possibly for survival leading to phenotypic differences for *Blastocystis.*

## References

[1] Windsor JJ (2007). Blastocystis hominis and Dientamoeba fragilis: neglected human protozoa. Br J Biomed Sci.

[2] Yoshikawa H, Abe N, Iwasawa M, Kitano S, Nagano I, Wu Z, Takahashi Y (2000). Genomic analysis of Blastocystis hominis strain isolated from two long-term health care facilities. Clinical Microbiology.

[3] Ustün S, Turgay N (2006). Blastocystis hominis and bowel diseases. Turkiye Parazitol Derg.

[4] Mansour NS, Mikhail EM, El Masrym NA, Sabry AG, Mohareb EW (1995). Biochemical characterization of human isolates of Blastocystis hominis. J Med Microbiol.

[5] Gericke A, Burchard G, Knobloch J, Walderich B (1972). Isoenzyme patterns of Blastocystis hominis patient isolates derived from symptomatic and healthy carriers. Trop Med Int Health.

[6] Kukoschke KG, Müller HE (1991). SDS-PAGE and immunological analysis of different axenic Blastocystis hominis strains. J Med Microbiol.

[7] Lanuza MD, Carbajal JA, Villar J, Mir A, Borrás R (1999). Soluble protein and antigenic heterogeneity in axenic Blastocystis hominis isolates: pathogenic implications. Parasitol Res.

[8] Müller H (1994). Four serologically different groups within the species Blastocystis hominis. Zentralbl Bakteriol.

[9] Böhm-Gloning B, Knobloch J, Walderich B (1997). Five subgroups of Blastocystis hominis isolates from symptomatic and asymptomatic patients revealed by restriction site analysis of PCR amplified 16S-like rDNA. Trop Med Int Health.

[10] Kaneda Y, Horiki N, Cheng XJ, Fujita Y, Maruyama M, Tachibana H (2001). Ribodemes of Blastocystis hominis isolated in Japan. Am J Trop Med Hyg.

[11] Yoshikawa H, Abe N, Wu Z (2004). PCR-based identification of zoonotic isolates of Blastocystis from mammals and birds. Microbiology-Engl Tr.

[12] Yan Y, Su S, Lai R, Liao H, Ye J, Li X, Luo X, Chen G (2006). Genetic variability of Blastocystis hominis isolates in China. Parasitol Res.

[13] Stensvold CR, Arendrup MC, Jespersgaard C, Mølbak K, Nielsen HV (2007). Detecting Blastocystis using parasitologic and DNA-based methods: a comparative study. Diagn Microbiol Infect Dis.

[14] Stensvold CR, Traub RJ, Von Samson-Himmelstjerna G, Jespersgaard C, Nielsen HV, Thompson RCA (2007). Blastocystis: subtyping isolates using Pyrosequencing™ technology. Exp Parasitol.

[15] Stensvold CR, Suresh GK, Tan KSW, Thompson RCA, Traub RJ, Viscogliosi E, Yoshikawa H, Clark CG (2007). Terminology for Blastocystis subtypes—a consensus. Trends Parasitol.

[16] Tan TC, Suresh KG, Smith HV (2008). Phenotypic and genotypic characterization of Blastocystis hominis isolates implicates subtype 3 as a subtype with pathogenic potential. Parasitol Res.

[17] Chan KH, Chandramathi S, Suresh K, Chua KH, Kuppusamy UR (2012). Effects of symptomatic and asymptomatic isolates of Blastocystis hominis on colorectal cancer cell line, HCT116. Parasitol Res.

[18] Yoshikawa H, Wu Z, Kimata I, Iseki M, Ali IK, Hossain MB, Zaman V, Haque R, Takahashi Y (2004). Polymerase chain reaction-based genotype classification among human Blastocystis hominis populations isolated from different countries. Parasitol Res.

[19] Hussein EM, Hussein AM, Eida MM, Atwa MM (2008). Pathophysiological variability of different genotypes of human Blastocystis hominis Egyptian isolates in experimentally infected rats. Parasitol Res.

[20] Wong KH, Ng GC, Lin RT, Yoshikawa H, Taylor MB, Tan KS (2008). Predominance of subtype 3 among Blastocystis isolates from a major hospital in Singapore. Parasitol Res.

[21] Özyurt M, Kurt Ö, Mølbak K, Nielsen HV, Haznedaroglu T, Stensvold CR (2008). Molecular epidemiology of Blastocystis infections in Turkey. Parasitol Int.

[22] Dogruman-Al F, Yoshikawa H, Kustimur S, Balaban N (2009). PCR-based subtyping of Blastocystis isolates from symptomatic and asymptomatic individuals in a major hospital in Ankara, Turkey. Parasitol Res.

[23] Souppart L, Sanciu G, Cian A, Wawrzyniak I, Delbac F, Capron M (2009). Molecular epidemiology of human Blastocystis isolates in France. Parasitol Res.

[24] Boorom KF, Smith H, Nimri L, Viscogliosi E, Spanakos G, Parkar U, Li LH, Zhou XN, Jones MS (2008). "Oh my aching gut: irritable bowel syndrome, Blastocystis, and asymptomatic infection.". Parasit Vectors.

[25] Malinen E, Rinttilä T, Kajander K, Mättö J, Kassinenm A, Krogiusm L, Saarela M, Korpela R, Palva A (2005). Analysis of the fecal microbiota of irritable bowel syndrome patients and healthy controls with real-time PCR. Am J Gastroenterol.

[26] Longstreth GF, Thompson WG (2006). Functional bowel disorders. Gastroenterology.

[27] Chaudhari HS, Singh PP (2010). Comparative drug susceptibility study of five clonal strains of Trichomonas vaginalis in vitro. Asia Pacific J Trop Med.

[28] Afzan MY, Sivanandam S, Kumar S (2010). Modified Field's staining-a rapid stain for Trichomonas vaginalis. Diagn Microbiol Infect Dis.

[29] Al FD, Hökelek M (2007). Is Blastocystis hominis an opportunist agent?. Turkiye Parazitol Derg.

[30] Stensvold CR, Lewis HC, Hammerum AM, Porsbo LJ, Nielsen SS, Olsen KEP (2009). Blastocystis: unravelling potential risk factors and clinical significance of a common but neglected parasite. Epidemiol Infect.

[31] Giannella RA, Broitman SA, Zamcheck N (1972). Gastric acid barrier to ingested microorganisms in man: studies in vivo and in vitro. Gut.

[32] Guyton AC, John EH (2006). Guyton and Hall Textbook of Medical Physiology with Student Consult Online Access.

[33] Bengmark S (1998). Ecological control of the gastrointestinal tract. The role of probiotic flora. Gut.

[34] Bonfrate L, Tack J, Grattagliano I, Cuomo R, Portincasa P (2013). Microbiota in health and irritable bowel syndrome: current knowledge, perspectives and therapeutic options. Scand J Gastroenterol.

[35] Noor SO, Ridgway K, Scovell L, Kemsley EK, Lund EK, Jamieson C, Johnson IT, Narbad A (2010). Ulcerative colitis and irritable bowel patients exhibit distinct abnormalities of the gut microbiota. BMC Gastroenterol.

[36] Joossens M, Huys G, Cnockaert M, DeP V, Verbeke K, Rutgeerts P, Vandamme P, Vermeire S (2011). Dysbiosis of the faecal microbiota in patients with Crohn's disease and their unaffected relatives. Gut.

[37] Sundin J, Rangel I, Hultgren-H¨ornquist E, Brummer R (2012). Increased number of double positive CD3+ CD8+ CD4+ lamina Propria T Lymphocytes in the gut mucosa of post-infectious IBS patients compare to healthy controls Rationale: Post infectious irritable bowel syndrome. Clin Nutr Suppl.

[38] Jones MS, Whipps CM, Ganac RD, Hudson NR, Boroom K (2009). Association of Blastocystis subtype 3 and 1 with patients from an Oregon community presenting with chronic gastrointestinal illness. Parasitol Res.

[39] Hameed DM, Hassanin OM, Zuel-Fakkar NM (2011). Association of Blastocystis hominis genetic subtypes with urticaria. Parasitol Res.

[40] Vinoth K, Umah RK, Suresh KG, Chandramathi S (2013). Blastocystis sp. subtype 3 triggers higher proliferation of human colorectal cancer cells, HCT116. Parasitol Res.

[41] Walderich B, Bernauer S, Renner M, Knobloch J, Burchard GD (1998). Cytopathic effects of Blastocystis hominis on Chinese hamster ovary (CHO) and adeno carcinoma HT29 cell cultures. Trop Med Int Health.

[42] Thompson RCA, Reynoldson JA, Mendis AHW (1993). Giardia and giardiasis. Adv Parasitol.

[43] Suresh K, Khairul AA, Saminathan T, Ng KP, Init I (1997). In vitro culture technique: a better diagnostic tool for Blastocystis hominis. J Int Med Res.

[44] Ma TY (1997). Intestinal epithelial barrier dysfunction in Crohn's disease. Biol Med.

[45] Barbara G, Zecchi L, Barbaro R, Cremon C, Bellacosa L, Marcellini M, De Giorgio R, Corinaldesi R, Stanghellini V (2012). Mucosal permeability and immune activation as potential therapeutic targets of probiotics in irritable bowel syndrome. J Clin Gastroenterol.

[46] Ohman L, Simrén M (2010). Pathogenesis of IBS: role of inflammation, immunity and neuroimmune interactions. Gastroenterol Hepatol.

[47] Olivo-Diaz A, Romero-Valdovinos M, Gudiño-Ramirez A (2012). Findings related to IL-8 and IL-10 gene polymorphisms in a Mexican patient population with irritable bowel syndrome infected with Blastocystis. Parasitol Res.

[48] Padilla-Vaca F, Anaya-Velázquez F (2010). Insights into *Entamoeba histolytica* virulence modulation. Infectious Disorders.

[49] Scanlan PD, Marchesi JR (2008). Micro-eukaryotic diversity of the human distal gut microbiota: qualitative assessment using culture-dependent and -independent analysis of faeces. ISME J.

[50] Ley RE (2008). Evolution of mammals and their gut microbes. Science.

[51] Rajilic SM, Smidt H, Willem MDV (2007). Diversity of the human gastrointestinal tract microbiota revisited. Environ Microbiol.

